# The promises and challenges of neurotechnology to improve human health and cognition

**DOI:** 10.1371/journal.pbio.3002903

**Published:** 2024-10-30

**Authors:** Simon Hanslmayr

**Affiliations:** 1 Centre for Neurotechnology, School of Psychology and Neuroscience, University of Glasgow, Glasgow, United Kingdom; 2 Centre for Cognitive Neuroimaging, School of Psychology and Neuroscience, University of Glasgow, Glasgow, United Kingdom

## Abstract

This *PLOS Biology* collection explores the present and possible futures of neurotechnology to improve human health and cognition, as well as the scientific, technological and ethical challenges they face.

Neurotechnology refers to devices that interface with the nervous system in order to augment or restore its function. The development of such devices is rapidly growing, with impressive progress in understanding how to interpret brain activity to drive external devices, or stimulate the brain to improve sensory or cognitive functions. Essentially, neurotechnology is the collaborative result of years of research in neuroscience, engineering, computer science, medicine, and philosophy. The first major breakthroughs in neuroscience were made in areas of basic sensory processing such as vision [1], hearing [2], touch and motor control [3]. It is therefore not surprising to see the first successful applications of neurotechnology also in these areas. This Issue of *PLOS Biology* features a collection of articles that examine the current state of such neurotechnologies, as well as their potential future applications and challenges.

Sensory feedback is often lost in neurological disorders; in their Perspective, Cimolato and Raspopovic take a closer look at neurotechnology that restores somatosensory feedback in patients and how this can enhance neurorehabilitation [4]. Concerning vision, we are witnessing the first applications of visual prostheses, which allow individuals with sight loss to perceive basic visual inputs. Although this is exciting news, significant technical and scientific challenges still need to be overcome before visual prostheses can be offered at scale, as discussed by Fernandez and Robles [5].

In contrast, our understanding of which brain mechanisms underly more complex functions such as attention, memory, decision making, or even consciousness, is much less developed. Despite this, actionable targets for neurotechnology have been identified, paving the way for their use not only for medical purposes, but also for enhancing neurocognition. In their Perspective, Violante and Okyere take a closer look at the potential for neurotechnology to revolutionize cognitive enhancement, focusing on the promise shown using non-invasive techniques [6]. And let’s not forget about memory; we have known for over 70 years that regions in the medial temporal lobe, most notably the hippocampus, are critical for memory. This area is routinely implanted with electrodes in patients suffering from epilepsy, which offers a direct pathway to invasively record from and stimulate these regions. Studies using such invasive methods are crucial for the development of memory prostheses which, similar to cochlear implants or visual prostheses, could restore a lost function such as memory in, for example, individuals with dementia. However, the findings in this area are rather mixed, with deep brain stimulation sometimes enhancing and sometimes impairing memory. Mohan and Jacobs discuss this issue and suggest potential avenues for the future, including individualized stimulation to obtain more consistent effects [7].

New technologies to interface with the brain are being developed at an increasing pace. For example, focused ultrasound stimulation, which has traditionally been used to create lesions by delivering high-intensity focal stimulation, can be used at low intensities to safely and reversibly stimulate the brain. In their Essay, Fouragnan and Murphy discuss the future of transcranial ultrasound stimulation and its potential to interface with the brain non-invasively at high precision [8]. Another exciting area is the use of quantum technology to enable high-precision brain-computer interfaces to be deployed ‘in the wild’, as discussed by Faccio [9]. However, these technologies need not be applied in isolation, but instead could be combined to maximise efficiency when interfacing with the brain, which is discussed in the Perspective by Micera and Foffani [10].

This *PLOS Biology* collection highlights that the future of neurotechnology is very exciting indeed, with novel and more powerful technologies to interface with the human brain at increasingly higher levels of precision and efficiency. However, such powerful techniques come with considerable ethical challenges, especially when they go beyond replacing basic sensory functions. In their Essay, Gordon and Seth discuss the ethical, legal and scientific issues raised by invasive brain-computer interfaces designed for human enhancement, and posit the fundamental and philosophical questions these raise [11].

As evidenced by these articles, neurotechnology is a highly interdisciplinary field where academia meets industry, and where neuroscientists, engineers, medics, psychologists and philosophers collaborate to drive research towards a common goal: To overcome the biological boundaries imposed by either disease or our own human limitations. Our aim with this article collection is to give an overview of the current state of neurotechnology, and to highlight open questions and important avenues for further research. We hope this collection inspires new ideas and fosters collaboration among diverse fields, ultimately advancing the potential of neurotechnology to improve human health and cognition.
